# Feasibility of Dialysate Bolus-Based Absolute Blood Volume Estimation in Maintenance Hemodialysis Patients

**DOI:** 10.3389/fmed.2022.801089

**Published:** 2022-02-10

**Authors:** Simon Krenn, Michael Schmiedecker, Daniel Schneditz, Sebastian Hödlmoser, Christopher C. Mayer, Siegfried Wassertheurer, Haris Omic, Eva Schernhammer, Peter Wabel, Manfred Hecking

**Affiliations:** ^1^Division of Nephrology and Dialysis, Department of Medicine III, Medical University of Vienna, Vienna, Austria; ^2^Department of Epidemiology, Center for Public Health, Medical University of Vienna, Vienna, Austria; ^3^AIT Austrian Institute of Technology, Center for Health & Bioresources, Medical Signal Analysis, Vienna, Austria; ^4^Division of Physiology, Otto Loewi Research Center, Medical University of Graz, Graz, Austria; ^5^Independent Researcher, Rosbach, Germany

**Keywords:** blood volume, chronic kidney disease, fluid status, hemodialysis, renal insufficiency, chronic, renal dialysis

## Abstract

**Background:**

Absolute blood volume (ABV) is a critical component of fluid status, which may inform target weight prescriptions and hemodynamic vulnerability of dialysis patients. Here, we utilized the changes in relative blood volume (RBV), monitored by ultrasound (BVM) upon intradialytic 240 mL dialysate fluid bolus-infusion 1 h after hemodialysis start, to calculate the session-specific ABV. With the main goal of assessing clinical feasibility, our sub-aims were to (i) standardize the BVM-data read-out; (ii) determine optimal time-points for ABV-calculation, “before-” and “after-bolus”; (iii) assess ABV-variation.

**Methods:**

We used high-level programming language and basic descriptive statistics in a retrospective study of routinely measured BVM-data from 274 hemodialysis sessions in 98 patients.

**Results:**

Regarding (i) and (ii), we automatized the processing of RBV-data, and determined an algorithm to select the adequate RBV-data points for ABV-calculations. Regarding (iii), we found in 144 BVM-curves from 75 patients, that the average ABV ± standard deviation was 5.2 ± 1.5 L and that among those 51 patients who still had ≥2 valid estimates, the average intra-patient standard deviation in ABV was 0.8 L. Twenty-seven of these patients had an average intra-patient standard deviation in ABV <0.5 L.

**Conclusions:**

We demonstrate feasibility of ABV-calculation by an automated algorithm after dialysate bolus-administration, based on the BVM-curve. Based on our results from this simple “abridged” calculation approach with routine clinical measurements, we encourage the use of multi-compartment modeling and comparison with reference methods of ABV-determination. Hopes are high that clinicians will be able to use ABV to inform target weight prescription, improving hemodynamic stability.

## Introduction

Fluid homeostasis is among the most complex physiological entities known to the medical sciences (1). It can become deranged in a variety of conditions such as intensive medical care (2, 3), cardiac failure (4, 5), and chronic kidney disease (CKD) (6, 7). Even in CKD patients not yet requiring kidney replacement therapy, chronic fluid overload is associated with increased mortality (7). Once CKD patients are on dialysis, optimal fluid management is essential for avoiding deleterious consequences at both ends of fluid dysbalance (i.e., fluid overload and excessive volume depletion) (8).

Restoration of the body's delicate electrolyte and water equilibria has been a perpetual quest of nephrologists from the 19 sixties onward and is the central goal of the common “dry weight” approach (6, 9–11). Clinical “dry weight,” originally defined as the target weight in a (hemo) dialysis (HD) patient at which the patient could not tolerate further fluid removal during the “probing dry weight” strategy, is not necessarily the same as the patient's euvolemic weight, determined by objective measures (9). Moreover, patients differ in their pathophysiological adoption of volume overload/depletion and susceptibility to fluid removal. Despite almost 60 years of HD experience, the physiological basis for fluid volume balance is unclear (12).

Blood volume monitoring (BVM) technology uses optical transmission/optical absorbance (13–16) or ultrasound (17–19) to measure the intradialytic concentration change of hemoglobin/hematocrit and to infer a relative change in blood volume from the hemoconcentration. The resulting BVM curve can be used to observe fluid content in the blood and thereby holds information on fluid status and optimal target weight, as steeper curves throughout HD indicate stronger intradialytic volume depletion (20). The original aim of intradialytic BVM, however, was to regulate the ultrafiltration (UF) rate based on the BVM signal to prevent intradialytic hypotension and related morbid events (21), thereby improving HD outcomes (22). In spite of some positive results regarding dialysis symptoms (23, 24), even higher mortality and hospitalization rates were initially observed with this technique (25), and the most recent large study that assessed hard outcomes was negative (12).

The main caveat of regulating the UF based on the intradialytic BVM curve during HD is that only relative blood volume (RBV) changes can currently be deduced from the BVM signal. These relative changes, however, are of little use when the absolute blood volume and its relation to the patient's overall volume status are unknown. Hecking and Schneditz compared the futility of controlling intravascular volume using only knowledge on RBV changes to a thermostat operating by temperature changes alone but ignoring the actual room temperature, which “could be anything” (21). Arguably, a measure for absolute blood volume (ABV), combined with bioimpedance-based extracellular volume assessment, could render an adapted RBV-guided UF beneficial, further enabling better explanation and control of blood pressure changes.

ABV can be measured using a variety of invasive, time-consuming methods, which are of little use in the clinical setting. Common methods range from radioactive tracer injection (26, 27) and CO-rebreathing (28, 29) to dye approaches [e.g., with indocyanine green (30, 31)]. Since 2014, Kron et al. published multiple articles on an abridged method to determine the patient's ABV during HD sessions (32–37). Utilizing the programmed “emergency function” of the Fresenius 5008 online hemodiafiltration machine (FMC, Bad Homburg, Germany) a bolus of 240 mL ultrapure dialysate was rapidly infused into the blood-stream (32). By manually reading the difference in RBV before and after this bolus administration directly from the screen of the dialysis machine, they approximated ABV and the specific blood volume in mL per kg body mass (specific blood volume, SBV) from the blood dilution caused by the injected fluid. Manual collection of the required data is too slow for clinical practice and prone to error and bias alike.

Thus, the aim of this study was to develop an automated algorithm to determine ABV by dialysate bolus (ABV-DB) from data habitually recorded by HD machines, implementing Kron et al.'s proposed method of calculation. To this end, we extracted and visualized the data recorded by the electronic interface, evaluated the correct implementation of this method in the clinical setting and assessed the intra-individual reproducibility of the resulting ABV-DB across multiple HD sessions in a cohort of CKD patients undergoing uninterrupted maintenance HD at a single tertiary care center.

## Methods

### Ethics Approval, Study Setting, and Participants

Boluses of ultrapure dialysate are fast and safe, and therefore an often-preferred alternative to intravenous fluid formulations during HD. At the Chronic Hemodialysis (CHD) Unit of the Vienna General Hospital, dialysate bolus administration for ABV determination was introduced into routine clinical practice as of September 2019. During this process, the targeted UF volume was increased to account for the added volume of the bolus. We obtained study approval from the Ethics Committee of the Medical University of Vienna (EC-No. 1732/2020, Project Title: *Closing the Loop in Hemodialysis: A Precision Medicine Approach – Part A [Intradialytic Determination of Absolute Blood Volume: An Exploratory, Retrospective Study on 98 Patients]*). The study adhered to the Declaration of Helsinki.

The CHD Unit of the Vienna General Hospital has a maximum capacity to treat 144 HD patients (thrice weekly) and is divided into two equally sized subunits, each one comprising 12 positions (HD slots) and executing 3 HD shifts per day. Various HD machines from 3 manufacturers (Fresenius, Nikkiso, Gambro) are in parallel use. Only the BVM-capable Fresenius 5008 was used for the dialysate bolus administration during the period of observation. Each CHD subunit was equipped with one such machine, which was moved from HD slot to HD slot as required. Each HD patient was scheduled to be studied during consecutive dialysis treatments with one bolus administered at every treatment. Patients undergoing hemodialysis or hemodiafiltration at the CHD Unit of the Vienna General Hospital, who had consecutive dialysate boluses for ABV-DB calculation scheduled from September to November 2019 and who did not require hospitalization between those HD sessions were assessed for eligibility.

### Data Retrieval and Visualization

All data collected by staff and HD machines (including the BVM data) were electronically stored in the hospital database by default. Nurses also routinely provided a short, informal report of each session, elaborating on irregularities and symptoms. Automatically recorded dialysis session data included BVM data, blood pressure (BP) and basic anthropometric patient information (sex, age, weight), and were extracted from the hospital database using the dialysis administration software Diamant 2 (Diasoft BV, Leusden, The Netherlands). Files containing these data were extracted for each patient using the Diamant system's individualizable reporting function. Laboratory data of quarterly routine blood work were extracted from the hospital database which operates with the clinical management software AKIM (SAP SE, Baden Württemberg, Germany). Data were parsed, pseudonymized and merged using Python 3.9 (Python Software Foundation, Beaverton, USA).

### Routine Blood Sampling

Blood was drawn from the patient's hemodialysis access, after discarding at least 10 mL in patients with venous catheters to avoid contamination with catheter lock solutions. Blood was always obtained prior to the HD session to rule out contamination with heparin used for the dialysis treatment. Blood was left to clot at room temperature and was transported to the central laboratory for analyses within 60–180 min after sampling.

### Blood Pressure

Systolic and diastolic BP were measured with the CHD Unit's standard BP cuffs (Philips Easy Care Adult M4555B) which are attached to the HD machines. BP measurements were triggered automatically at standard 1-h intervals, or additionally, as clinically needed. To avoid artifacts caused by the bolus injection and white coat effect, BP data collected during the dialysate bolus application itself were analyzed separately.

### Absolute Blood Volume Determination

The ABV-DB at the beginning of the dialysis was determined as described in Equation (1) (32, 38):


(1) $$\begin{array}{l} \text{AB}{\text{V}}_{\text{DB},0}\left(\text{mL}\right)=\frac{{\text{V}}_{\text{DB}}\;\left(\text{mL}\right)}{\text{RB}{\text{V}}_{\text{after}}\left(\text{\%}\right)-\text{RB}{\text{V}}_{\text{before}}\left(\text{\%}\right)}\times \text{RB}{\text{V}}_{0}\left(\text{\%}\right) \end{array}$$

*RBV*_*before*_ was defined as the last recorded RBV value before bolus injection, *RBV*_*after*_ as the maximum RBV value within a 15-min interval after bolus injection and *RBV*_0_ as the first RBV value recorded during HD. *V*_*DB*_ represented the volume of the dialysate bolus, in our case 240 mL as used in most publications on this method (34). Only bolus administrations of 240 mL ultrapure dialysate between 50 and 120 min after dialysis start were eligible for analysis. If RBV data expected within a 15-min window before or after bolus were missing, or if an injection of 240 mL was not completed within 3 min [considering that the average infusion time was 1 min 56 ± 20 s standard deviation [SD]], the corresponding electronic record was excluded from the study as well.

### Statistical Evaluation

Descriptive statistics, specifically means with SDs for normally and medians with interquartile ranges (IQRs) for non-normally distributed data were used to present patient characteristics, HD, and laboratory data (Tables 1–3). RBV curves were visualized using line plots (**Figures 2A,B**), and blood pressure data were depicted in box-and-whisker plots (**Figure 2C**). For a statistical measure of ABV-DB reproducibility, we used the average intra-patient SD of ABV-DB (**Figure 3A**) on a range of plausible data sampling cut-offs. These cut-offs define the time windows from which the RBV values for the ABV-DB calculations are drawn. These windows always started at the bolus (bolus start or completion, respectively) and ranged back or forward in time for up to 15 min (as shown on the horizontal axes). Average intra-patient SD is a type of statistic which exhibits lower values with increasing similarity of ABV-DB values across multiple HD sessions within the same patient.

**Table 1:** Patient characteristics (based on 86 patients).

**Patient characteristics**		***N* = 86**
Age (years), mean (SD)		58.6 (16.5)
Sex, *n* (%)	Female	33 (38.4)
	Male	53 (61.6)
Height (cm), mean (SD)		169.3 (9.9)
Weight before dialysis (kg), mean (SD)		72.8 (15.2)
Target weight (kg), mean (SD)		70.8 (15.2)
BMI before dialysis (kg/m^2^), mean (SD)		25.4 (4.7)
Access type, *n* (%)	Catheter	33 (38.4)
	Shunt	53 (61.6)
Residual diuresis (mL), median [Q1, Q3]		325.0 [0.0, 800.0]
Diuresis below 200 mL/day, *n* (%)	No	49 (57.0)
	Yes	37 (43.0)
Creatinine (mg/dL), mean (SD)		9.6 (3.1)
Diabetes, *n* (%)	No	66 (76.7)
	Yes	20 (23.3)
HbA1c (%), median [Q1, Q3]		5.2 [4.8, 5.6]
Glucose (mg/dL), median [Q1, Q3]		102.0 [90.8, 117.0]
CRP (mg/dL), median [Q1, Q3]		0.6 [0.2, 1.4]
Ferritin (μg/L), median [Q1, Q3]		395.8 [193.5, 573.7]
Transferrin (mg/dL), median [Q1, Q3]		169.0 [144.0, 197.0]
Transferrin saturation (%), median [Q1, Q3]		20.9 [14.7, 28.2]
Hematocrit (%), mean (SD)		30.8 (3.8)
Hemoglobin (g/dL), mean (SD)		10.2 (1.3)
Erythrocytes (G/L), mean (SD)		3.4 (0.5)
Sodium (mmol/L), mean (SD)		139.4 (3.6)
Chloride (mmol/L), mean (SD)		99.4 (4.5)
Potassium (mmol/L), mean (SD)		5.3 (0.7)
Calcium (mmol/L), median [Q1, Q3]		2.2 [2.0, 2.3]
Inorganic phosphate (mmol/L), median [Q1, Q3]		1.9 [1.4, 2.5]
Parathyroid hormone (pg/mL), median [Q1, Q3]		298.7 [134.5, 494.1]
Urea (mg/dL), mean (SD)		64.7 (20.1)
Uric acid (mg/dL), mean (SD)		6.7 (1.4)
Total bilirubin (mg/dL), median [Q1, Q3]		0.3 [0.2, 0.4]

*SD, Standard Deviation; Q1, First Quartile; Q3, Third Quartile*.

**Table 2:** Fluid status, weight and blood pressure (based on 86 patients, 186 sessions).

	**Measurements (*n*)**	**Mean**	**SD**	**Minimum**	**25%**	**Median**	**75%**	**Maximum**
UF volume (ml)	186	2490.0	1132.7	10.0	1676.0	2440.0	3404.5	4800.0
Target weight (kg)	180	71.1	15.1	38.0	62.5	68.3	81.0	115.0
Weight before dialysis (kg)	181	73.1	15.1	38.9	65.0	70.6	83.8	115.9
Weight after dialysis (kg)	162	72.1	15.3	38.4	63.0	68.8	82.4	115.0
Intradialytic ABV-DB reduction (L)	186	−0.4	0.6	−1.7	−0.6	−0.4	−0.2	6.0
Intradialytic RBV reduction (%)	186	−8.4	7.5	−31.7	−13.0	−7.0	−3.4	5.5
IDWL (kg)	162	−2.0	1.1	−4.5	−2.9	−2.0	−1.1	0.3
IDWG (kg)	162	1.9	1.3	−4.2	1.1	1.8	2.7	6.6
Systolic BP before dialysis (mmHg)	172	137.0	23.3	83.0	121.0	136.0	153.0	216.0
Systolic BP after dialysis (mmHg)	145	130.9	25.9	73.0	113.0	132.0	151.0	189.0
Diastolic BP before dialysis (mmHg)	172	69.4	17.2	14.0	59.8	68.0	80.0	160.0
Diastolic BP after dialysis (mmHg)	145	67.8	16.3	26.0	58.0	69.0	79.0	128.0
Systolic BP reduction (mmHg)	139	6.5	23.2	−53.0	−8.0	8.0	18.0	119.0
Diastolic BP reduction (mmHg)	139	2.6	15.1	−53.0	−5.0	1.0	8.0	100.0
Duration of dialysis (H:M:S)	186	03:53:47	00:31:38	02:02:16	03:33:57	04:00:26	04:10:46	05:21:09

*ABV-DB, Dialysate bolus derived blood volume; UF, Ultrafiltration; RBV, Relative blood volume; IDWL, Intradialytic weight loss; IDWG, Interdialytic weight gain; UF, Ultrafiltrate; BP, Blood pressure; H, Hours; M, Minutes; S, Seconds*.

**Table 3:** ABV-DB (based on 75 patients, 145 sessions).

	**Measurements (*n*)**	**Mean**	**SD**	**Minimum**	**25%**	**Median**	**75%**	**Maximum**
ABV-DB start of dialysis (L)	145	5.1	1.5	2.3	4.2	5.0	5.8	10.5
Nadler's BV before dialysis (L)	141	4.7	0.8	2.7	4.1	4.7	5.3	6.7
Nadler's BV target (L)	140	4.6	0.8	2.6	4.1	4.7	5.2	6.7
SBV Start of dialysis (ml/kg)	141	72.2	23.7	28.8	54.5	66.6	85.6	144.8
RBV before bolus (%)	145	95.7	4.0	85.4	93.1	96.0	98.6	104.8
RBV after bolus (%)	145	100.7	3.8	90.7	97.4	101.3	103.3	109.5
RBV delta caused by bolus (%)	145	5.0	1.4	2.3	4.1	4.8	5.7	10.5
RBV end of dialysis (%)	145	92.3	7.1	68.7	88.9	93.5	96.8	105.5
RBV peak delay (M:S)	145	06:14	02:16	02:00	04:56	05:34	07:04	16:32

*ABV-DB, Dialysate bolus derived bolus volume; RBV, Relative blood volume; BV, Blood Volume; BP, Blood Pressure; SD, Standard Deviation; H, Hours; M, Minutes; S, Seconds; RBV peak delay denotes the time passed from bolus injection start to the maximum RBV value within 15 min after completion of the bolus injection (this includes the bolus duration ≤ 3 Min, hence maxima over 15 min are possible)*.

Concerning the data sampling time window before and after the bolus for the ABV-DB calculation, obviously, more RBV data points became available with greater sampling time windows and, accordingly, it would be more likely to find both a before-, as well as an after-bolus RBV value, which are both necessary for the ABV-DB calculation. Thereby, the number of curves valid for calculation increases with sampling time window size (**Figure 3B**).

For each patient with more than one RBV curve, the SD between ABV-DB estimates was calculated over all possible sampling cut combinations (which are defining the time intervals before and after bolus from which we sampled the RBV values for ABV-DB calculation) within 0 to 15 min before and 0 to 15 after bolus injection, with a granularity of 0.3 s. These intra-patient SDs were averaged over all patients for each sampling cut combination (before- and after-bolus) and then visualized using 3D surface plots (Note that, setting other constraints aside, the optimal sampling cut combination would arguably be the pair of time points before and after bolus, at which the average intra-patient SDs of ABV-DB are minimal, as this cut combination on average results in the most consistent ABV-DB estimation within patients. First and foremost, however, this type of plot is suited to show sampling cut combinations that produce strong disagreement between the estimates, which clearly should be avoided.) All analyses, figures and tables were computed using Python 3.9 (Python Software Foundation, Beaverton, USA).

## Results

### Patient Flow and Characteristics

**Figure 1:**
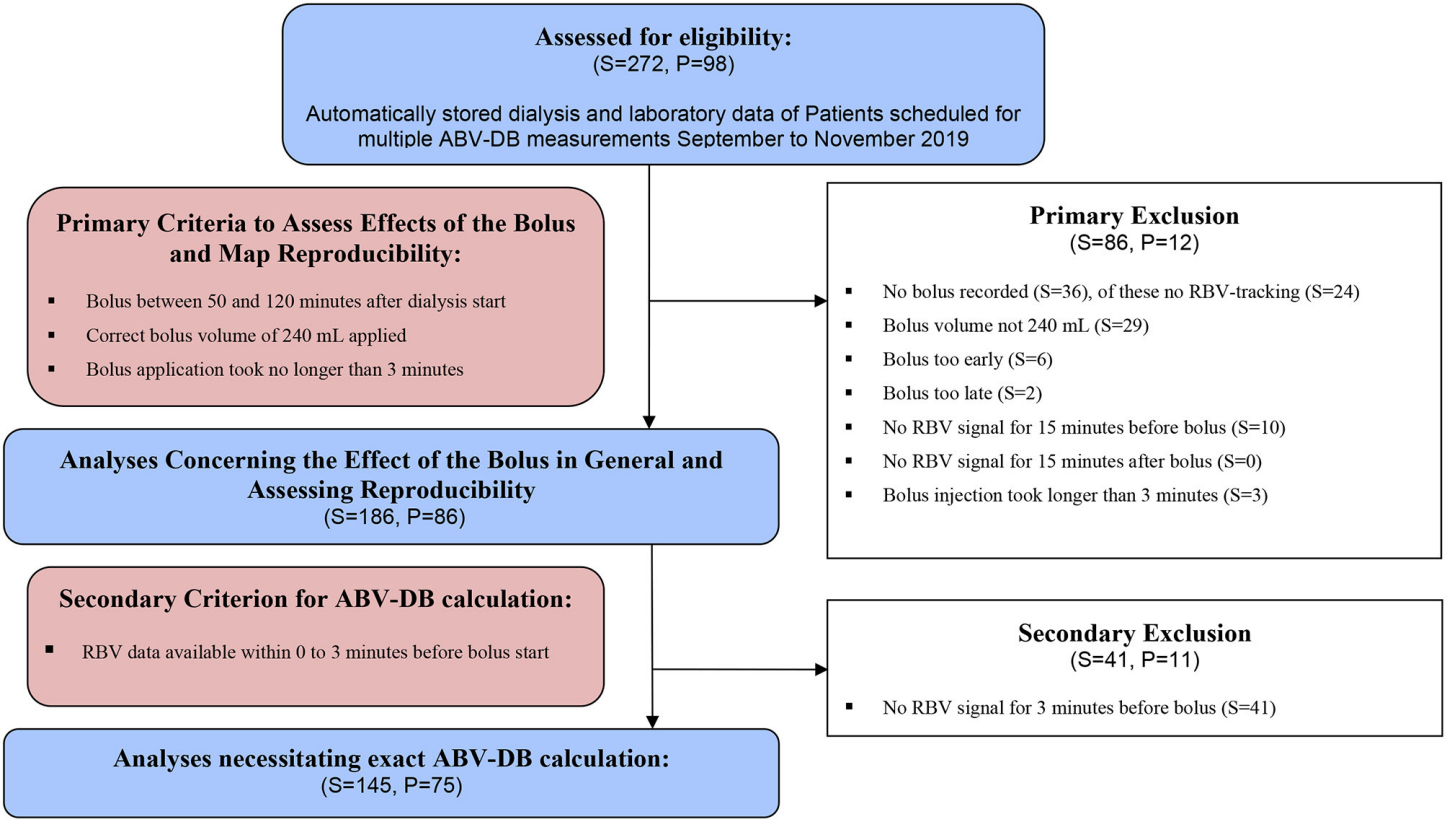
Patient flow chart. S, Sessions; P, Patients.

The present analysis was separated into two parts with different exclusion criteria. For the first part we excluded 86 BVM curves from 13 patients (details of the exclusion criteria are mentioned in the Methods and reported in Figure 1, Primary Exclusion). The characteristics of these 86 patients are shown in Table 1. The average age of the patients was 58.5 ± 16.6 years, and the mean weight was 72.6 ± 15.2 kg. Patients were on average slightly overweight at a mean body mass index (BMI) of 25.3 ± 4.7 kg/m^2^, and 23.3% were diabetic. HD was applied via the central venous catheter access prevalence in women was actually 57.6% (Supplementary Table 1).

For the second part of the analysis, we excluded 44 additional BVM curves from 10 patients, because no RBV data were available for 3 min before the bolus was injected (Figure 1, Secondary Exclusion). The ABV-DB calculations were therefore based on the remaining 145 BVM curves in 75 patients.

### BVM Curve Visualization and Blood Pressure

**Figure 2:**
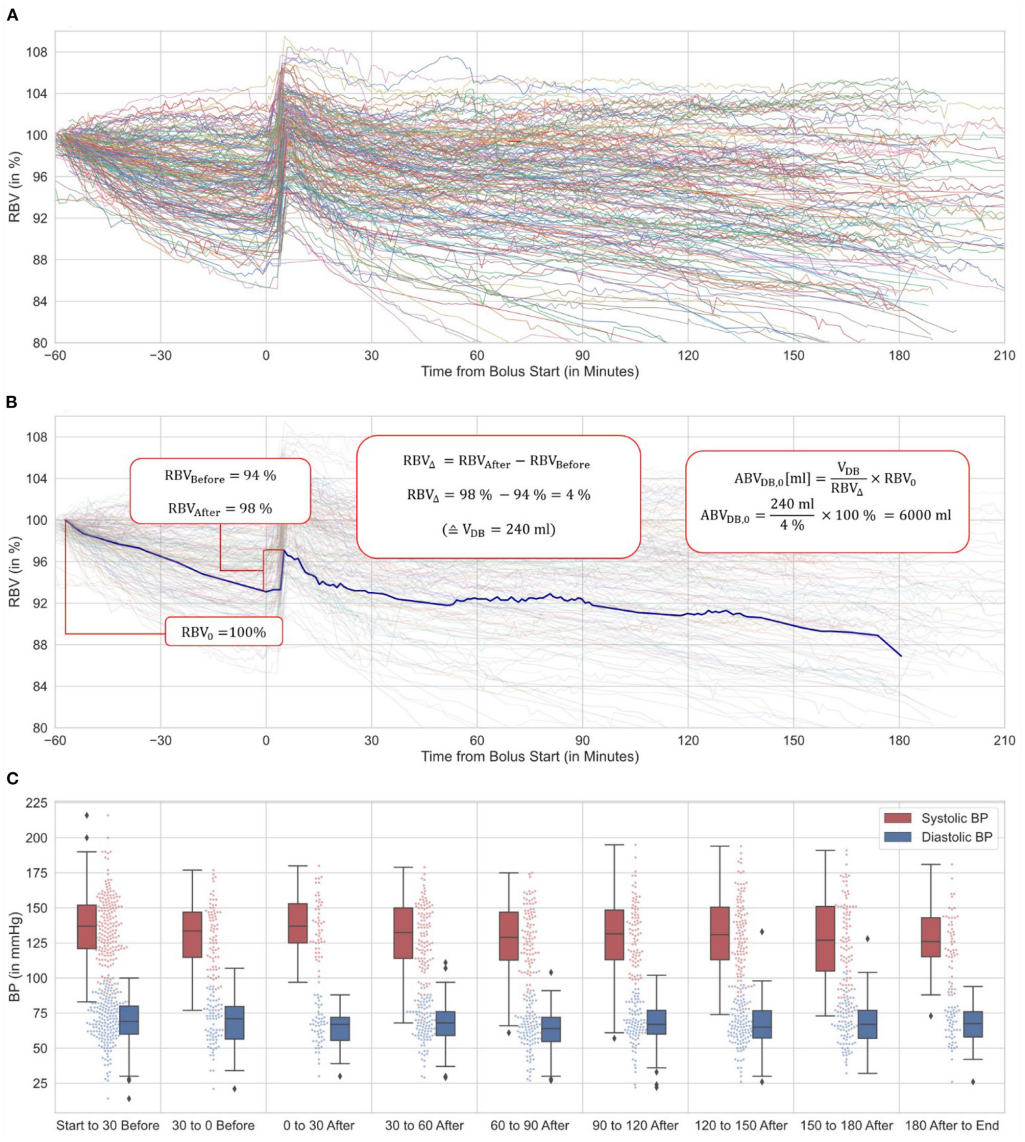
BVM-curve variation, ABV-DB calculation details and blood pressure (based on 86 patients, 186 sessions). **(A)** 186 RBV curves showing significant divergence in curve morphology and individual progression after bolus administration. **(B)** Examplary RBV curve and ABV-DB calculation using dialysate bolus method. **(C)** Systolic and diastolic blood pressure over time relative to the dialysate bolus are depicted in box-and-whisker and jitter plots. BPs during the bolus application are omitted.

The BVM curves of 186 complete HD sessions (primary exclusion criteria applied) are shown in Figure 2A. At the time of dialysate bolus injection (t = 0), a resulting spike in RBV was observable. The example of a BVM curve over a complete dialysis session is shown in Figure 2B, describing the entity of the bolus, and depicting also the exact points on an exemplary BVM curve where RBV values were extracted for the calculation of ABV-DB. The blood pressure values of all patients over time are provided in Figure 2C. We observed a narrowing of both systolic and diastolic blood pressure during the first 30 min after the bolus (boxes and whiskers). Notably, no patient exhibited systolic BP below 90 mmHg during this time.

### Setting the Window for RBV Extraction

**Figure 3:**
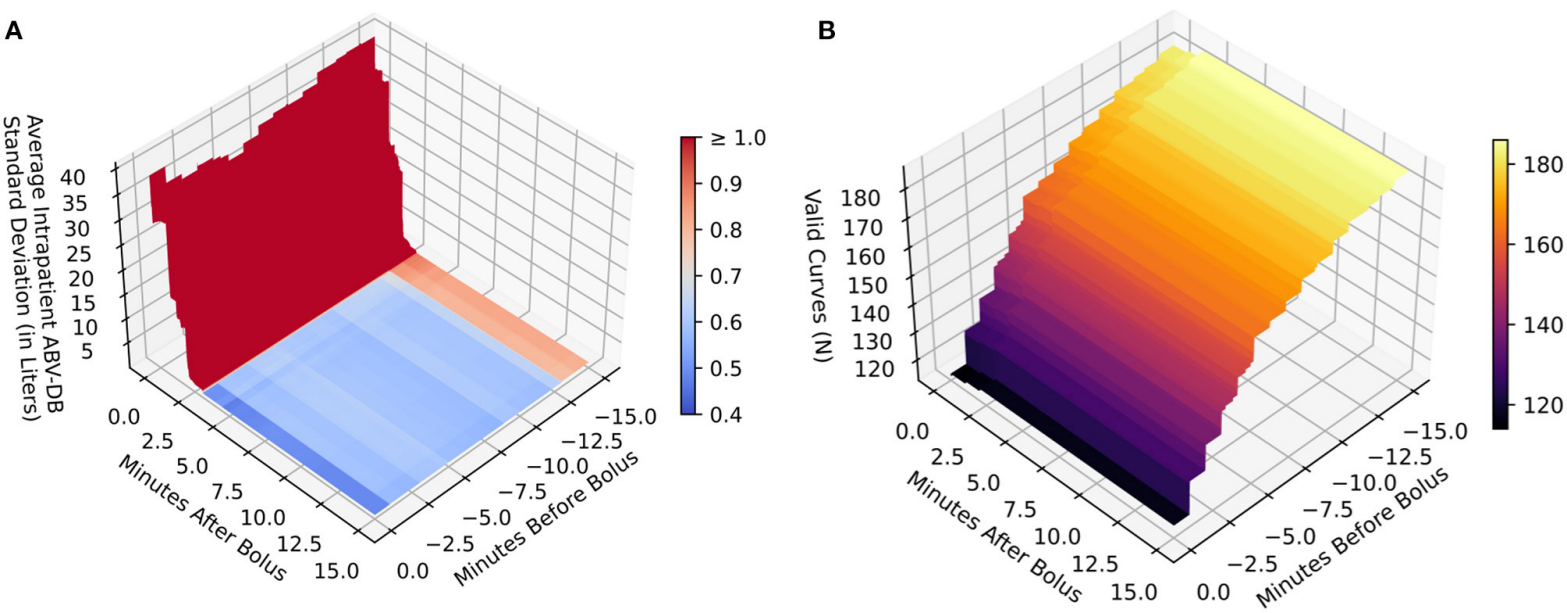
Reproducibility and calculability mapping of all patients with at least two valid measurements (based on 86 patients, 186 sessions). **(A)** Reproducibility between measurements measured by intra-patient standard deviation of the ABV-DB (vertical axis *Z*) by time cut-offs around bolus (horizontal axes *X* and *Y*). **(B)** Amount of curves usable for ABV-DB calculation (vertical axis *Z*) by time cut-offs around bolus (horizontal axes *X* and *Y*). Color bars in **(A,B)** relate to values on the respective vertical axes *Z*. This figure maps out the reproducibility and calculability of ABV-DB by the two-point calculation method as previously used by Kron et al. when applying different time interval cut-offs from bolus for the inclusion of data. This serves to find cut-offs that produce robust results on average and do not exclude too many curves, but further methodological considerations should also inform decisions on choosing the correct cut-off intervals. Only data on the RBV curves within the cut-off interval range is used for calculations of the corresponding *Z*-axis values on these graphs. For the range before bolus, the last measured RBV value before the bolus is used. The reason, why more curves produce usable results with increasing interval size is that in some curves the last measured RBV value is outside the cut-off interval if it is too small. As the interval size is increased, these values become available for analysis and hence the number of valid curves increases. For the range after bolus, the maximum RBV value is used. A reliable RBV maximum for ABV-DB calculation occurs after the dialysate bolus is adequately distributed in the blood stream (at least 2.5 min after completion of the bolus injection) and remains robust to further interval increases thereafter.

There was a need to limit the time window from which the RBV values before and after bolus were drawn for the calculation of the RBV amplitude. Otherwise, the last RBV value before bolus would have been too long before bolus to deliver reliable estimates of ABV, and the maximum RBV value after bolus might have been falsely high, due to higher local maxima not related to the bolus, and encountered at a later time point during the HD session. The range of possible data sampling time-cuts from bolus and the corresponding average intra-patient SDs of ABV-DB are shown in Figure 3A. The corresponding numbers of usable curves at the respective settings are reported in Figure 3B. As seen in Figure 3A, the sampling window after bolus clearly needed to be >2.5 min, but provided a robust variable for ABV-DB calculation thereafter. For the sampling cut before bolus, only small changes were observed in the ABV-DB deviation between estimates, but many calculations became impossible (because data were scarcer here) when this time-cut was set too close to the bolus, as seen in Figure 3B.

We also observed a clear trade-off between the theoretical validity of the method and the number of usable BVM curves. As the time interval of the sampling cut before bolus was increased, more BVM curves delivered calculable results. Specifically, as more RBV values were encountered in both the before and after bolus intervals, the BVM curve was included as a “valid curve” in Figure 3B. However, allowing later recorded values to enter the calculation could have also led to unreliable ABV-DB estimates. In our case, if the last 3 min before bolus had generally contained no RBV data, changes in RBV would on average have resulted in an absolute difference ± SD of 0.86 ± 2.55 liters of blood. As this estimation error was expected to be cumulative over time (leading on average to an inflated ABV-DB), there was a need to collect RBV values as closely before the bolus injection as possible.

We concluded that the optimal time points for RBV extraction for Kron's abridged ABV-DB calculation method were the last measured RBV value before the bolus and the maximum RBV within 15 min after the bolus. Note that the rationale here above justifiably prompted the exclusion of those BVM curves where no RBV data were available between 3 and 0 min before bolus start (44 curves in 10 patients, as mentioned in the first paragraph of the Results Section and shown in Figure 1), from analyses requiring correct ABV-DB estimates.

### Resulting ABV-DB

HD-specific variables are shown in Table 2 and blood volume data (including ABV-DB and SBV) are shown in Table 3. We observed a very wide range of ABV-DB between patients (mean 5.2 ±1.5 L). In 64.1 % of cases, blood volume after bolus was actually greater than at the start of dialysis.

**Figure 4:**
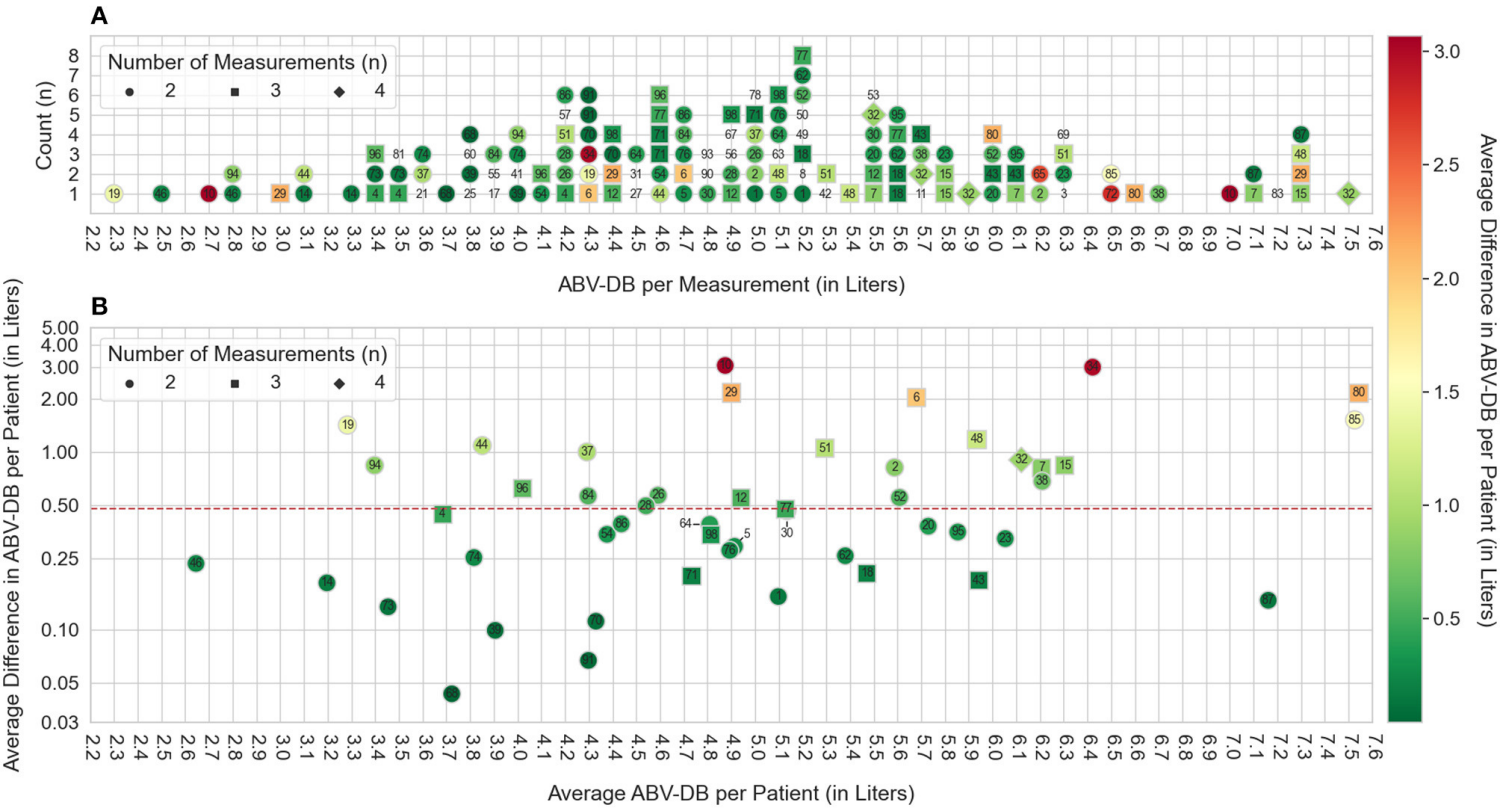
ABV-DB distribution and standard deviation (based on 75 patients, 145 sessions). **(A)** Stacked dot-plot assessing ABV-DBs between 2.2 and 7.3 liters (70 Patients, 116 Sessions fall within this cut-off). Numeric patient identifiers are annotated for each ABV-DB estimate. The vertical axis shows the count of estimates which fall within a given 0.1 L interval of ABV-DB. Coloring of the annotation represents the average difference in ABV-DB observed in the respective patient, as specified in the color bar to the right of the figure: Green denotes high agreement between estimates, red denotes poor agreement. Patients without a colored contour marker did not have a second ABV-DB estimate available for comparison and therefore their average difference of ABV-DBs could not be calculated. **(B)** The horizontal axis here denotes the average ABV-DB for the corresponding patient. Coloring of annotations is analogous to **(A)** The vertical axis is on a logarithmic scale and denotes the average difference between ABV-BD estimates observed within the patient and is analogous to the coloring of the annotation. Patient 5, 30, and 64 were annotated with an offset to improve readability. The median average intra-patient difference in ABV-DB for all patients is denoted by a red dashed line.

The distribution of ABV-DB between 2.2 and 7.6 liters, as well as a color-scaled measure of agreement between multiple estimates within patients, if available, are shown in a stack plot histogram in Figure 4A. Edge cases of very high ABV-DBs were not included in this graph, but their BVM curves and theories on why these BVM curves resulted in ABV-DB outliers are discussed in the Supplementary Material. The variation of the ABV-DB between estimates, depending on the average value of ABV-DB, is shown on a logarithmic scale for improved visibility in Figure 4B. Patients with the highest values on the average ABV-DB scale exhibited higher variation between estimates.

## Discussion

Here we employed a previously published method to estimate the ABV by applying an intradialytic bolus of ultrapure dialysate. In our understanding, the present study adds important information to the existing literature, not only by representing a larger population size than the earlier reports (32–37, 39), but also because repeated measurement sessions per patient were performed, and possible pitfalls regarding insufficient sampling rates using an automatic data acquisition systems were fully disclosed. To evaluate the method, we made an effort to use as much data as possible for each analysis, implementing separate, but genuine exclusion steps, as necessary for validity.

ABV-DB determination using data collected in the routine clinical setting proved feasible. Nevertheless, we found that many BVM curves had to be excluded due to technical problems in the RBV sampling rate, but some also due to divergent execution in the clinical setting (e.g., non-standard bolus volume). We were able to formalize the approach for calculation provided by Kron et al. using an automated algorithm and extracting the BVM curves form the dialysis machines with a dialysis administration software. This approach is expected to provide an unbiased analysis compared to manual and direct visual inspection of data.

The manual approach described elsewhere was introduced for clinical use in want of a suitable electronic data acquisition system. Data acquisition systems recording all relevant machine data from whole dialysis units are currently not designed for data collection at high sampling rates. In our case data sampling was transmitted at increments of whole minutes, which is less than optimal. However, this sampling rate was not achieved consistently in some cases, which we expect to hold true for other standard interface systems as well. If the specified sampling period of 1 min were adequately maintained, it would still remain difficult to identify the proper concentrations required for the two-point method. In fact, analyzing the time course preceding and following the bolus dilution might be necessary. The abridged two-point approach inherently assumes instantaneous and stable distribution of indicator and a simple step change in concentrations, with stable (or steadily changing) concentrations before and after dilution. This assumption is of course idealized as blood concentrations are very variable during *in vivo* dialysis, when recorded with the precision required for BVM purposes. Nevertheless, the estimation of plausible concentrations could for example be improved by time series analysis of data points, regarding their variability and trend, and extrapolating the series of pre- and post-dilution concentrations to the time of dilution and the time of complete mixing, respectively.

Our results showed a high variability in ABV-DB, namely an average intra-patient SD of 0.78 L (median SD 0.47 L) in the 51 patients who had undergone at least two valid measurement sessions. In a quarter of repeat estimates, the intra-patient SD was 0.26 L or lower. Whether the observed variation is (at least partially) due to actual changes in blood volume between HD sessions or due to inaccuracy of the applied method cannot be determined without comparison to accepted reference methods, such as indocyanine green or radioisotope-based measurements. In future studies, we might also be able to assess whether cumulative information from a high number of BVM curves can reduce the intra-patient SD.

BP measurements performed during the dialysis sessions notably showed no drops below 90 mmHg systolic in the half-hour after bolus administration. Hospital staff also received informal feed-back from some patients who reported a positive subjective effect on overall wellbeing during and after HD sessions involving a bolus for ABV-DB estimation. As low BP and intradialytic hypotension are risk factor for outcomes, in future it might be interesting to assess the clinical outcomes of patients who receive a dialysate bolus at every dialysis session in comparison to those of a control group (even without informing dry weight adjustment or guided ultrafiltration). Whether patient-reported outcomes are purely due to placebo effect, or have some hard physiological correlate, could be an interesting side topic for future investigations applying this method. Evidence for the beneficial effect of repeated bolus infusion as used during intermittent back-filtrate infusion hemodiafiltration in reducing intradialytic hypotensive events is limited, but seems promising and could be a welcome side effect in measuring ABV-DB as well (40–42).

Concerning improved HD safety, Kron et al. observed no intradialytic morbid events, above 65 mL/kg SBV in a study encompassing 45 HD patients (33). This proposed static threshold requires further examination in larger cohorts and should probably be adapted depending on patient and treatment characteristics, as a volume-per-mass approach may be overly simplistic especially in obese subjects. We observed that an occasionally occurring sampling gap before bolus may lead to more unreliable ABV-DB estimates. For example, if RBV had not been recorded during the last 3 min before bolus in our study collective, this lack would have on average resulted in an absolute difference in ABV-DB estimation of 0.86 liters of blood with a high SD of 2.55 liters. It is therefore advisable to ensure a high sampling rate for electronic data transfer from the dialysis monitor to the data acquisition system before applying the fluid bolus.

Our study collective appeared largely representative of a standard hemodialysis population. However, a high number of patients (38.4%) received dialysis through a central catheter. This type of HD access was more prevalent in women and might have led to different results, due to the more central location of the catheter. The difference between catheters and fistulas regarding ABV-DB determination should receive special attention by investigators in the future. Especially when using a multi-compartment modeling approach, access type may be an important point to consider during model specification. In our own study ABV-DB (and SBV) was on average only 121.8 mL (1.4 mL/kg) lower in HD sessions involving a catheter access. In female patients who had a central catheter access it was 301.8 mL (8.41 mL/kg) lower, but in male patients 146.1 mL (4.3 mL/kg) higher. This result requires confirmation from future studies, and whether the location of the catheter might be causal currently remain purely speculative.

To improve accuracy and physiological plausibility, more complex models should probably be used, taking into account the intravascular/extravascular/interstitial fluid shifts. In this vein, Samandari et al. have recently published a study comparing a two compartment model to a back-extrapolation method (39). While not explicitly addressed, a main figure in their paper shows that ABV-DB values were consistently estimated lower in patients with a central catheter, using either of the modeling approaches they employed. Important points to consider during modeling include the cardiopulmonary recirculation, fluid shifts between the intravascular and interstitial spaces, as well as changes in the distribution of hematocrit between central and peripheral blood volume compartments. Future studies should also compare ABV-DB calculations with accepted reference methods [e.g., with indocyanine green (30, 31)].

In conclusion, we acknowledge the high variability in ABV-DB and a number of unreliable estimates due to a lack of BVM curve stability as the principal study limitation. Nevertheless, BVM data extraction and processing for ABV-DB calculation proved feasible. Further improvement might be made by increasing the sampling rate in the data acquisition system and by applying more sophisticated models of the cardiovascular space including whole body fluid distribution and kinetics. Establishing accuracy and reproducibility, ideally by receiving direct help from HD machine manufacturers now is the most important subsequent step along the way of enabling clinicians to use ABV-calculations such as to inform target weight prescription. Hopes are high that hemodynamic stability may be improvable once ABV estimates become more reliable and the dynamic relationship with the overall fluid status is elucidated.

## Data Availability Statement

The raw data supporting the conclusions of this article will be made available by the authors, without undue reservation.

## Ethics Statement

The studies involving human participants were reviewed and approved by Ethics Committee of the Medical University of Vienna (EC No. 1732/2020, Project Title: Closing the Loop in Hemodialysis: A Precision Medicine Approach – Part A [Intradialytic Determination of Absolute Blood Volume: An Exploratory, Retrospective Study on 98 Patients]). Written informed consent for participation was not required for this study in accordance with the national legislation and the institutional requirements.

## Author Contributions

SK designed the study, collected data, analyzed data, and wrote the manuscript. MS and HO collected data and reviewed the manuscript. DS and PW designed the study, discussed the results, and reviewed and corrected the manuscript. SH designed the study and discussed the analysis. CM, SW, and ES discussed the results and reviewed and corrected the manuscript. MH designed the study, discussed the results, wrote the manuscript, and reviewed and corrected the manuscript. All authors contributed to the article and approved the submitted version.

## Funding

This work was supported by the Vienna Science and Technology (WWTF) Grant LS20-079 Precision Medicine.

## Conflict of Interest

The authors declare that the research was conducted in the absence of any commercial or financial relationships that could be construed as a potential conflict of interest.

## Publisher's Note

All claims expressed in this article are solely those of the authors and do not necessarily represent those of their affiliated organizations, or those of the publisher, the editors and the reviewers. Any product that may be evaluated in this article, or claim that may be made by its manufacturer, is not guaranteed or endorsed by the publisher.

## Abbreviations

ABV: absolute blood volume
ABV-DB: absolute blood volume by dialysate bolus method
BP: blood pressure
BVM: blood volume monitoring
CHD: chronic hemodialysis
CKD: chronic kidney disease
HD: hemodialysis
KDIGO: Kidney Disease Improving Global Outcomes
r: Pearson's correlation coefficient
RBV: relative blood volume
SD: standard deviation
UF: ultrafiltration.
