# Supplementary material for: Feasibility of Dialysate Bolus-Based Absolute Blood Volume Estimation in Maintenance Hemodialysis Patients
Source: Front Med (Lausanne). 2022 Feb 10;9:801089. doi: 10.3389/fmed.2022.801089 (PMC8866453; doi:10.3389/fmed.2022.801089)
Supplement: Supplementary file 1 [file Presentation_1.pdf]

Supplementary Tables and Figures

		Missing	Overall	Female	Male
N			86	33	53
Age (years), mean (SD)		0	58.6 (16.5)	62.4 (16.1)	56.3 (16.5)
Sex, n (%)	Female	0	33 (38.4)	33 (100.0)	
	Male		53 (61.6)		53 (100.0)
Height (cm), mean (SD)		0	169.3 (9.9)	160.5 (5.6)	174.8 (7.8)
Weight Before Dialysis (kg), mean (SD)		0	72.8 (15.2)	66.1 (13.4)	76.9 (14.9)
Target Weight (kg), mean (SD)		1	70.8 (15.2)	63.7 (13.0)	75.3 (14.9)
BMI Before Dialysis (kg/m²), mean (SD)		0	25.4 (4.7)	25.6 (5.1)	25.2 (4.5)
Access Type, n (%)	Catheter	0	33 (38.4)	19 (57.6)	14 (26.4)
	Shunt		53 (61.6)	14 (42.4)	39 (73.6)
Residual Diuresis (mL), median [Q1,Q3]		0	325.0 [0.0,800.0]	300.0 [0.0,700.0]	350.0 [0.0,800.0]
Diuresis below 200 mL/day, n (%)	No	0	49 (57.0)	20 (60.6)	29 (54.7)
	Yes		37 (43.0)	13 (39.4)	24 (45.3)
Creatinine (mg/dL), mean (SD)		1	9.6 (3.1)	8.6 (2.7)	10.2 (3.3)
Diabetes, n (%)	No	0	66 (76.7)	25 (75.8)	41 (77.4)
	Yes		20 (23.3)	8 (24.2)	12 (22.6)
HbA1c (%), median [Q1,Q3]		7	5.2 [4.8,5.6]	5.0 [4.7,5.4]	5.2 [5.0,5.7]
Glucose (mg/dL), median [Q1,Q3]		2	102.0 [90.8,117.0]	97.0 [86.0,116.0]	106.0 [92.5,117.5]
CRP (mg/dL), median [Q1,Q3]		0	0.6 [0.2,1.4]	0.6 [0.2,1.0]	0.4 [0.2,1.4]
Ferritin (μg/L), median [Q1,Q3]		3	395.8 [193.5,573.7]	336.2 [181.2,530.3]	404.1 [196.7,645.1]
Transferrin (mg/dL), median [Q1,Q3]		3	169.0 [144.0,197.0]	170.0 [156.5,188.5]	169.0 [142.0,198.5]
Transferrin Saturation (%), median [Q1,Q3]		3	20.9 [14.7,28.2]	16.6 [13.6,25.7]	22.8 [14.9,30.1]
Hematocrit (%), mean (SD)		0	30.8 (3.8)	31.0 (3.7)	30.6 (3.9)
Hemoglobin (g/dL), mean (SD)		0	10.2 (1.3)	10.1 (1.2)	10.2 (1.3)

**Erythrocytes (G/L), mean (SD)**	0	3.4 (0.5)	3.5 (0.5)	3.4 (0.5)
**Sodium (mmol/L), mean (SD)**	1	139.4 (3.6)	139.3 (3.6)	139.4 (3.6)
**Chloride (mmol/L), mean (SD)**	1	99.4 (4.5)	99.3 (4.3)	99.4 (4.6)
**Potassium (mmol/L), mean (SD)**	1	5.3 (0.7)	5.3 (0.8)	5.3 (0.6)
**Calcium (mmol/L), median [Q1,Q3]**	0	2.2 [2.0,2.3]	2.2 [2.1,2.3]	2.1 [2.0,2.3]
**Inorganic Phosphate (mmol/L), median [Q1,Q3]**	0	1.9 [1.4,2.5]	1.9 [1.6,2.5]	1.9 [1.3,2.5]
**Parathyroid Hormone (pg/mL), median [Q1,Q3]**	4	298.7 [134.5,494.1]	176.8 [83.3,362.1]	333.2 [212.9,567.3]
**Urea (mg/dL), mean (SD)**	0	64.7 (20.1)	63.4 (21.0)	65.5 (19.7)
**Uric Acid (mg/dL), mean (SD)**	2	6.7 (1.4)	6.5 (1.2)	6.9 (1.6)
**Total Bilirubin (mg/dL), median [Q1,Q3]**	1	0.3 [0.2,0.4]	0.3 [0.2,0.3]	0.3 [0.3,0.5]

Supplementary Table 1: Patient Characteristics by Gender (based on 86 Patients). SD: Standard Deviation, Q1: First Quartile, Q3: Third Quartile

	Patients (n)	Mean	SD	Minimum	25%	Median	75%	Maximum
**UF Volume (mL)**	86	2478,5	1099,0	10,0	1725,0	2509,3	3285,8	4745,0
**Target Weight (kg)**	84	70,6	15,2	38,0	62,4	68,8	81,0	115,0
**Weight Before Dialysis (kg)**	85	72,5	15,3	38,9	64,1	70,6	84,1	115,7
**Weight After Dialysis (kg)**	80	71,3	15,2	38,4	62,4	68,8	82,1	115,0
**Intradialytic ABV-DB Reduction (L)**	86	-0,4	0,4	-1,2	-0,6	-0,4	-0,2	1,3
**Intradialytic RBV Reduction (%)**	86	-8,6	7,0	-29,3	-13,5	-7,0	-3,6	3,7
**IDWL (kg)**	80	-2,0	1,1	-4,3	-2,7	-1,9	-1,2	0,0
**IDWG (kg)**	80	1,9	1,1	-1,2	1,0	1,8	2,6	4,7
**Systolic BP Before Dialysis (mmHg)**	83	136,9	21,7	88,0	123,3	135,7	152,2	190,0
**Systolic BP After Dialysis (mmHg)**	78	130,3	25,4	73,0	113,3	132,4	150,0	182,5
**Diastolic BP Before Dialysis (mmHg)**	83	69,7	15,6	33,5	58,7	70,0	81,8	115,5
**Diastolic BP After Dialysis (mmHg)**	78	67,7	17,0	26,0	60,1	69,0	78,9	128,0
**Systolic BP Reduction (mmHg)**	77	6,0	20,3	-53,0	-5,0	7,7	17,0	58,0
**Diastolic BP Reduction (mmHg)**	77	2,3	13,2	-53,0	-5,3	2,0	8,0	52,0
**Duration of Dialysis (H:M:S)**	86	03:52:57	00:29:44	02:36:29	03:35:14	03:59:55	04:08:31	05:17:42

Supplementary Table 2: Mean Patient Fluid Status, Weight and Blood Pressure (based on 86 Patients, 186 Sessions): ABV-DB, Dialysate bolus derived blood volume, UF: Ultrafiltration, RBV: Relative blood volume, IDWL: Intradialytic weight loss, IDWG: Interdialytic Weight Gain, UF: Ultrafiltrate, BP: Blood pressure, H: Hours, M: Minutes, S: Seconds

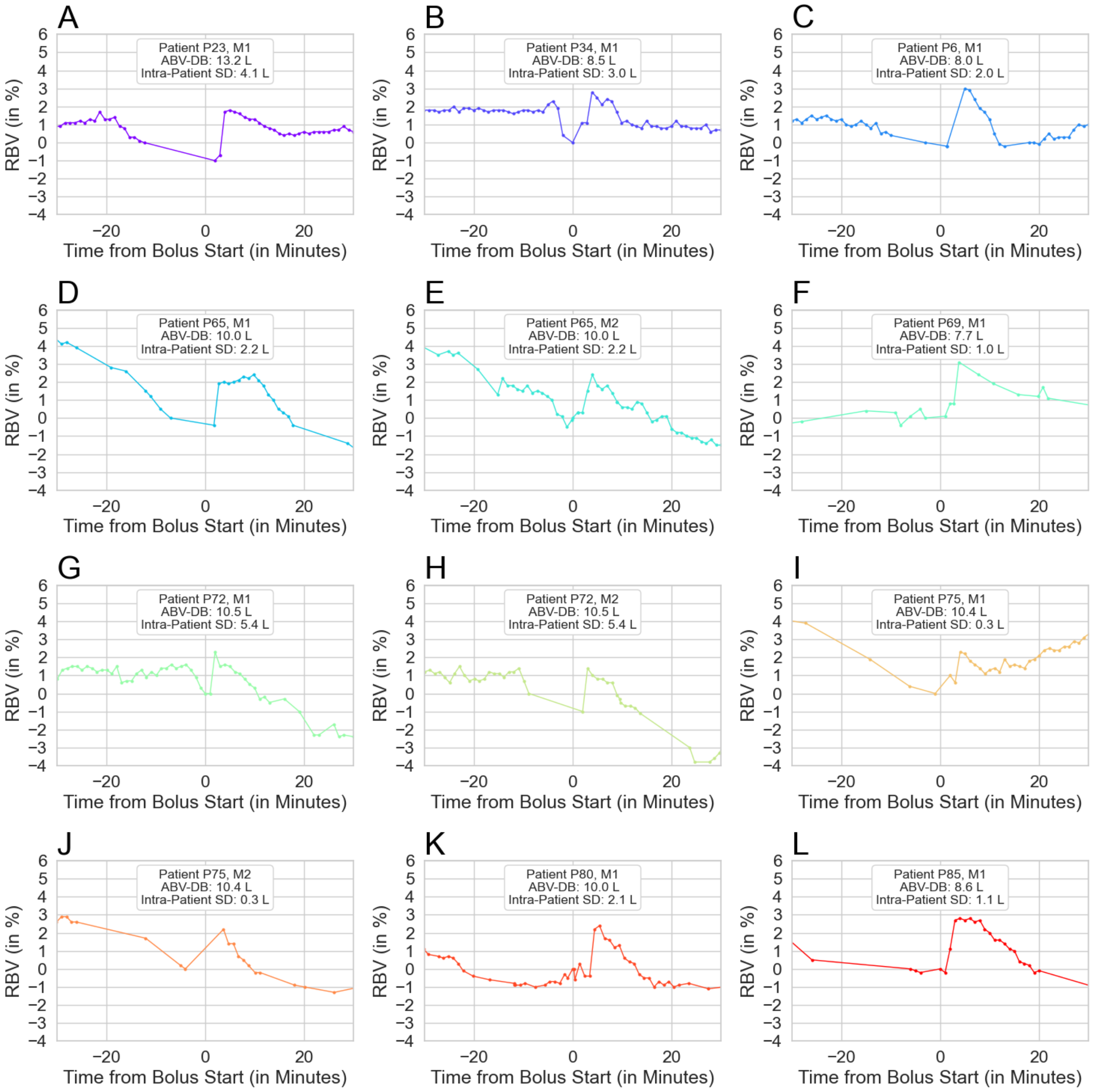

Supplementary Figure 1: Close-Up around Bolus of all BVM Curves with ABV-DB $\geq$ 7.6 Liters (based on 86 Patients, 186 Sessions).

Y-axis is zeroed to the RBV value at bolus application time. Measured data points are marked as dots on the curves. Colors Correspond to the same patients as in **Supplementary Figure 2**. In some curves a relatively steep decline in RBV can be observed before the bolus application, which may lead to falsely high ABV-DB estimates using this method (**Panels B, E, G**). The standard deviation in some patients is high and suggests this to be an erratic measurement (**Panels G and H**). In others the SD is low, indicating that the ABV-DB, while abnormally high, was reproducible in this patient (**Panels I and J**). In some patients there was a sampling gap (**Panels A, D, F, H, J**) of more than 3 minutes before the bolus application, which leads to unreliable estimates and exclusion from ABV-DB analyses (see **Results** section for explicit explanation). In two patients no plausible reason for high ABV-DB estimates was apparent via inspection the BVM curve alone (**Panels C, K, L**). Note that this analysis uses all 186 HD sessions of exclusion step 1 (see **Figure 1**) as a basis for SD calculations and therefore does not correspond to **Figure 4** and **Table 3**.

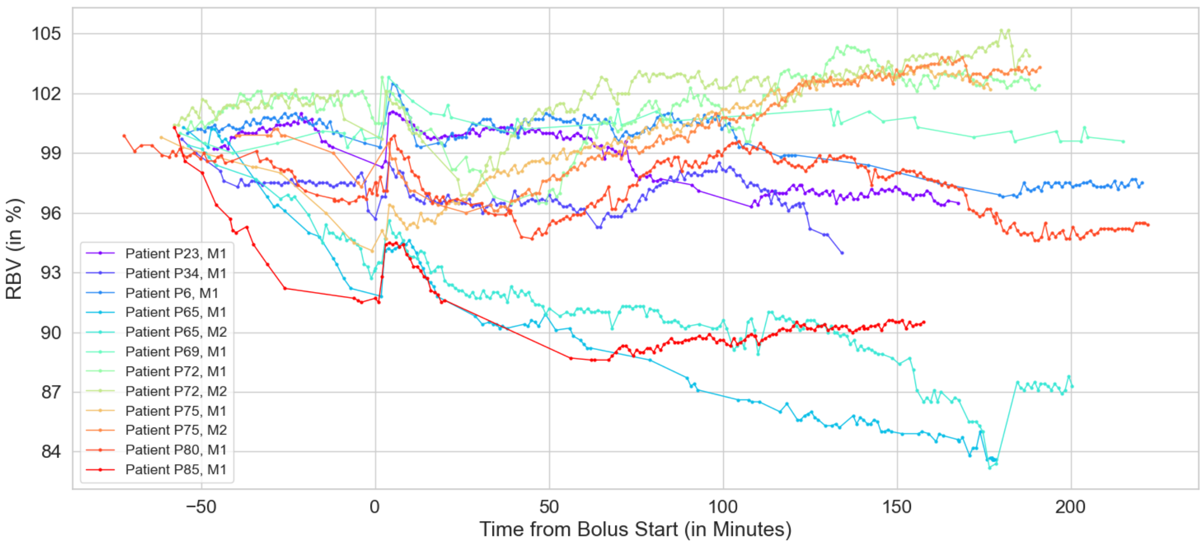

Supplementary Figure 2: BVM Curves with ABV-DB ≥ 7.6 Liters (based on 86 Patients, 186 Sessions).
Measured data points are marked as dots on the curves. Colors Correspond to the same patients as in **Supplementary Figure 1**. Curves with a high ABV-DB do not show a uniform pattern of RBV reduction over time.
